# Modelling the Impact of Robotics on Infectious Spread Among Healthcare Workers

**DOI:** 10.3389/frobt.2021.652685

**Published:** 2021-05-25

**Authors:** Raul Vicente, Youssef Mohamed, Victor M. Eguíluz, Emal Zemmar, Patrick Bayer, Joseph S. Neimat, Juha Hernesniemi, Bradley J. Nelson, Ajmal Zemmar

**Affiliations:** ^1^ Department of Neurosurgery, Henan Provincial People’s Hospital, Henan University People’s Hospital, Henan University School of Medicine, Zhengzhou, China; ^2^ Institute of Computer Science, University of Tartu, Tartu, Estonia; ^3^ Instituto de Física Interdisciplinar y Sistemas Complejos IFISC (CSIC-UIB), Palma de Mallorca, Spain; ^4^ Department of Neurosurgery, University of Louisville, School of Medicine, Louisville, KY, United States; ^5^ Multi-Scale Robotics Laboratory, Swiss Federal Institute of Technology (ETH) Zurich, Zurich, Switzerland

**Keywords:** robotics, COVID-19, epidemiology, healthcare, biomedical robots

## Abstract

The Coronavirus disease 2019 (Covid-19) pandemic has brought the world to a standstill. Healthcare systems are critical to maintain during pandemics, however, providing service to sick patients has posed a hazard to frontline healthcare workers (HCW) and particularly those caring for elderly patients. Various approaches are investigated to improve safety for HCW and patients. One promising avenue is the use of robots. Here, we model infectious spread based on real spatio-temporal precise personal interactions from a geriatric unit and test different scenarios of robotic integration. We find a significant mitigation of contamination rates when robots specifically replace a moderate fraction of high-risk healthcare workers, who have a high number of contacts with patients and other HCW. While the impact of robotic integration is significant across a range of reproductive number R_0_, the largest effect is seen when R_0_ is slightly above its critical value. Our analysis suggests that a moderate-sized robotic integration can represent an effective measure to significantly reduce the spread of pathogens with Covid-19 transmission characteristics in a small hospital unit.

## Introduction

The Coronavirus disease 2019 (Covid-19) pandemic has had a devastating impact on global healthcare and economy. The rapid global spread of the Severe Acute Respiratory Syndrome Coronavirus (SARS-CoV-2) is owed to its high transmissibility (van Doremalen et al., 2020), transmission prior to symptom onset (Tindale et al., 2020), and infectious spread through asymptomatic carriers (Bai et al., 2020). These features have posed significant challenges in various sectors, especially essential services such as the healthcare sector. Several measures are taken to reduce infectious spread for patients and healthcare worker (HCW) protection while maintaining healthcare services (The Lancet, 2020). Nevertheless, infection rates of up to 20% among HCW are reported in certain countries (Remuzzi and Remuzzi, 2020). As of October 2020, 7,000 HCW have died of Covid-19 worldwide (https://www.amnesty.org/en/latest/news/2020/09/amnesty-analysis-7000-health-workers-have-died-from-covid19/), of which 1,077 deaths and almost 80,000 HCW positive cases have been confirmed within the United States (https://www.washingtonpost.com/graphics/2020/health/healthcare-workers-death-coronavirus/), where various infection control measures including a lockdown, social distancing and personal protective equipment (PPE) have been implemented. In another study, 50% of HCW have reported their work setting as the single source of exposure (Burrer et al., 2020). Among patients, the elderly generation in geriatric units and nursing homes has been primarily affected by the pandemic. Prolonged close contact between patients and HCW, e.g., assistance in patient care and daily needs such as eating, bathing, walking or lifting, are among the prevailing causes that expose the elderly community and their caregivers to increased risk of contamination (McMichael et al., 2020).

The use of robotics as a shielding layer between patients and caregivers is a promising approach to reduce infectious spread in healthcare (Yang et al., 2020). While some hospitals have started to use robotic technology to combat the pandemic and one ward was entirely staffed by robots (O’Meara, 2020), modelling data in real-hospital scenarios is lacking to investigate the efficiency and timing of robotic implementation for reduction of pathogen contamination (Heesterbeek et al., 2015). Here, we utilized a temporally and spatially precise dataset of close personal contacts of 29 patients and 46 HCW in a geriatric unit (Vanhems et al., 2013). We identify nodes of high contact representing increased contagion risk, replace these high-risk nodes and model contamination rates. The model demonstrates that strategically placed robotic assistance to high-risk HCW can significantly reduce and delay the number of infections in a hospital unit in diseases with similar transmissibility to Covid-19. In the studied scenario, robotic integration is shown to be effective across a wide range of reproductive number R_0_ from slightly above 1 to at least 4.4.

## Results

### Effects of Robotic Replacement of Healthcare Workers

**FIGURE 1:**
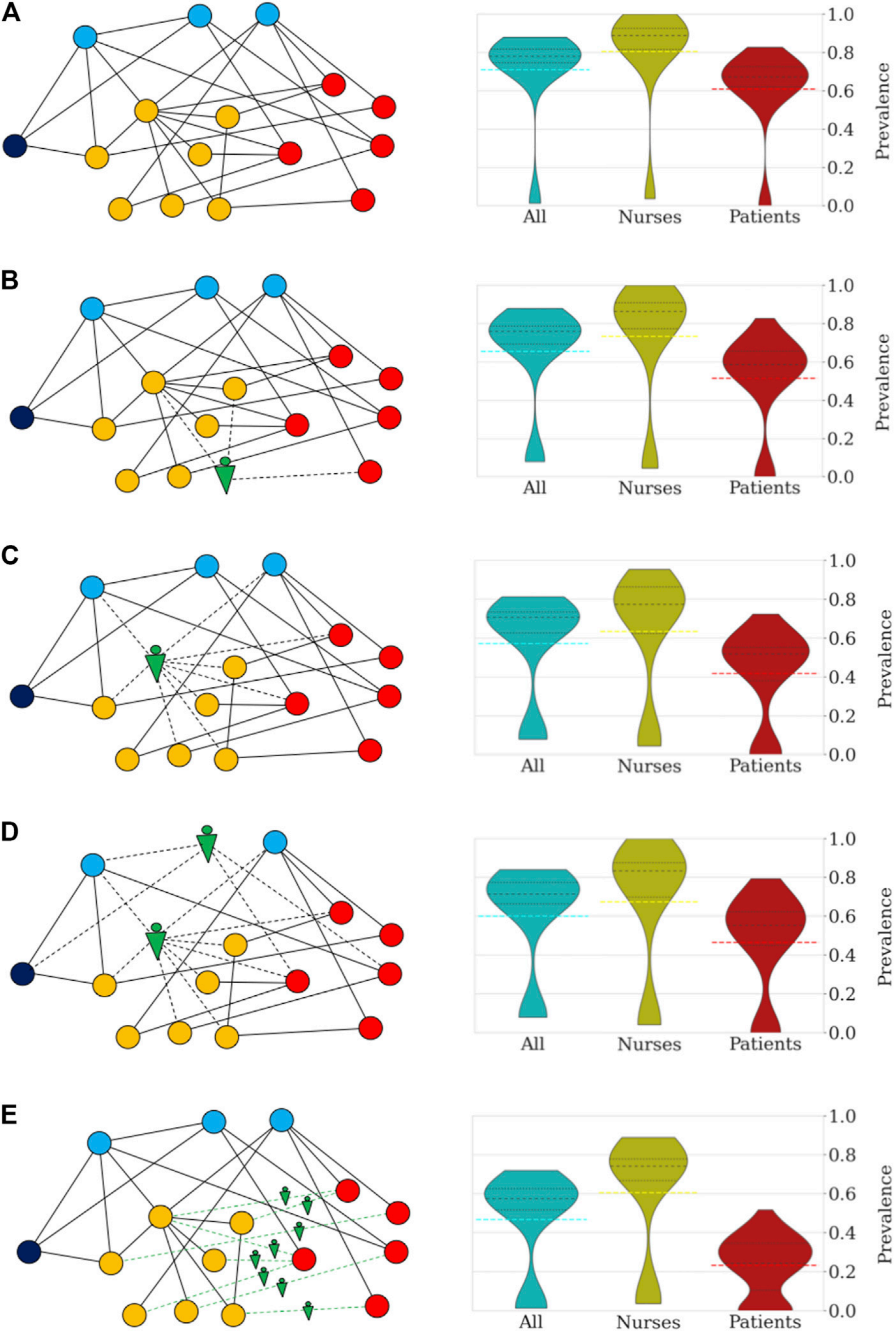
Robotization scenarios and their impact on the probability of pathogen spread through the network and number of infected individuals (R_0_ = 4.4). The networks on the left side depict sketches of the networks of close contacts at the geriatric ward under different scenarios. The colors indicate the categories: patients (red), nurses (orange), doctors (blue), administration (dark blue), and robots (green). **(A)** In scenario (i) no robotic assistance is provided and the network of close contacts remains intact. **(B)** Scenario (ii) is illustrated by a random nurse being replaced by a robot. **(C)** In scenario (iii) high-risk nurses are being replaced. **(D)** Scenario (iv) is illustrated by the robotic replacement of a high-risk nurse and a random doctor. **(E)** In scenario (v) the robotization affects the interactions between nurses and patients. The right side of each panel shows the distribution of prevalence of infection (percentage of individuals infected) under the different scenarios. All simulations were repeated 100 times to obtain the distributions shown in the violin plots and percentile statistics. For each violin plot, the dashed black lines indicate the different quartiles of the distribution while dashed color lines mark the mean. **(A)** In the absence of robotic intervention, the distribution of the number of infected cases is bimodal with 10% of trials in which the infection does not spread. Across all trials, an average of 71% of the personnel and patients become eventually infected. In general, the nurses and doctors are more vulnerable to becoming infected than patients and administrative workers. **(B)** Random replacement of five nurses increases the percentage of non-spreading trials to 16%. This intervention has the effect of decreasing the average number of infected individuals (66%). **(C)** The situation improves further when the five nurses are selected according to the number of contacts. In this case, the probability that the infection does not spread increases to 21% (more than doubling the case without robotization). Patients and nurses benefit from the targeted intervention with an increase in the number of non-infected cases when the infection spreads. On average, the fraction of infected individuals decreases to 57%. **(D)** Replacement of high-risk nurses and random doctors resulted in a similar probability of no propagation through the network (22%), while resulting in an average of 60% infected individuals. **(E)** Interaction replacement led to a probability that the infection does not spread in 23% of the trials, and an average of only 47% of individuals being infected. For patients the impact is most significant with the majority of simulations predicting that less than 25% of patients of the geriatric unit become eventually infected.

Robots can be utilized to assist HCW in a variety of tasks (Supplementary Table S1) and hence, to reduce the number and duration of interactions among different types of HCW and patients. To effectively mitigate pathogen contamination and operate cost-efficient, it is critical to determine high-risk groups of individuals and interactions and deploy robotic assistance specifically to these nodes. In a previous study, nurse-to-patient interactions accounted for 21.1% and nurse-nurse interactions resulted in 39.2% of the total number of close contacts (>20 s and <1.5 m) in a geriatric unit (Vanhems et al., 2013). Data from the same study shows that five nurses were responsible for 36.1% of all close contacts with patients. Based on these observations, we focused on replacing nurses with robots and simulate pathogen transmission in a geriatric unit with a model tailored to the state, transmissibility, and latency of SARS-CoV-2 (Ivorra et al., 2020; Eguíluz et al., 2020). Infectious spread was modelled under five scenarios: (i) no robotic assistance, and four scenarios in which robots replaced five random nurses (ii), the top five high-risk nurses (iii), the top 3 high-risk nurses and 2 random medical doctors (iv) and finally, when robots replaced all interactions between nurses and patients (v) (Figure 1). The model was run for 100 trials, results were averaged. Each simulation describes the pathogen spreading over the network and timing of close contacts in a geriatric unit, while we collect statistics of the transmission for the curse of an outbreak, which often lasted more than 90 days. A non-spreading state was defined when less than 10% of the individuals become infected within a single trial, whereas a spreading state was defined when more than 10% of individuals become infected. Without use of robotic technology, only 10 of 100 trials (10%) resulted in a non-spreading case. (Figure 1A). The probability of a non-spreading dynamics is augmented by 6% (i.e. from 10 to 16%) if five random nurses are replaced by robots (Figure 1B). In scenarios iii, iv and v, a strategic process was used by first identifying the nurses with the highest number of contacts (i.e. high-risk nurses) and selectively replacing them with robots. When robots replaced the five high-risk nurses (iii) (Figure 1C), the probability to contain the virus was 22%, or in other words, the probability of non-spreading dynamics increased by 110% when compared to the baseline scenario (i) without any robotic assistance (from 10 to 22%). To compare the impact of the five high-risk nurses with other medical staff, we replaced the top 3 high-risk nurses and 2 random medical doctors (MD) with robots, which resulted in a 21% probability to contain the virus (scenario iv, Figure 1D). In the last scenario, robotic assistance was applied to all interactions between nurses and patients, i.e., no nurse had direct contact with a patient, but nurses can still interact with other staff. This measure resulted in a 23% probability not to spread the pathogen (Figure 1E). Detailed numerical results of these and other measures of infection are collected in Supplementary Table S2.

### Temporal Evolution of Infectious Spread

**FIGURE 2:**
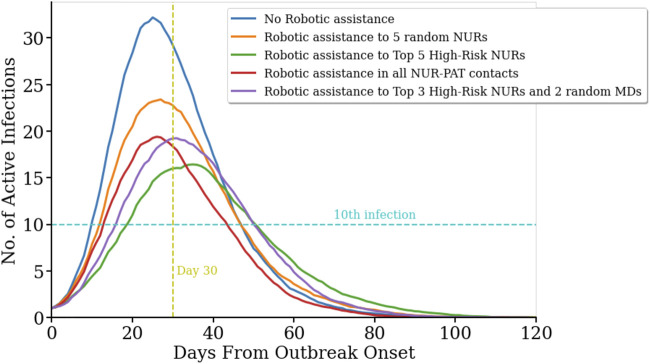
Temporal evolution of infection spread across the network for difference scenarios.(R_0_ = 4.4). Number of active infected cases in five different scenarios as a function of the days passed since the first infection in the population on day zero. No robotic integration (blue line) yields in the fastest onset and highest number of active infected cases. In contrast, robotic replacement of the five nurses with the highest number of contacts with other Health Care Workers (HCW) and patients results in the slowest increase and the overall lowest number of active cases (green line). Replacing five random nurses with robots leads to more peak active cases and a steeper slope (orange curve). Replacing all nurse-to-patient contacts (red line) and the top three high-risk nurses and two random medical doctors (purple line) resulted in a similar peak for active cases.

The temporal evolution of the infectious spread is also a key aspect for the management of an outbreak. For example, the speed of propagation and the peak number of active infected cases are important challenges to the limited reaction time and capacity for a response to the outbreak. Here, we focus on how pathogen contamination propagates across the network in the geriatric unit and describe how the different robotic assistance scenarios affect the temporal dynamics of the infectious spread. We measured the number of days from outbreak onset until the 10th infection occurs (T_10_), and the number of infections on the 30th day from outbreak onset (I_30_). As observed in Figure 2, no robotic assistance leads to the highest and earliest peak in the number of infected cases, with T_10_ = 10.1 ± 0.7 days (average ± s.e.m., *n* = 100), and I_30_ = 32.5 ± 0.8 active infected cases (average ± s.e.m., *n* = 100). Robotic replacement of the top five high-risk nurses leads to a significant delay of 1 week to reach the 10th infection in the population (T_10_ = 17.1 ± 0.8 days, n = 100; p_val_ = 0, permutation test for statistical difference with T_10_ without robotic assistance), and also results in a significant reduction of the number of active infected cases by day 30 (I_30_ = 19.2 ± 1.0 cases, *n* = 100; p_val_ = 0, permutation test for statistical difference with I_30_ without robotic assistance). Strikingly, this scenario shows both, the slowest initial growth of infection propagation and the slowest final decay, and hence it produces the largest flattening of the curve of active infections (Figure 2, green line). Robotic replacement of all nurse-patient interactions results in the earliest termination of the outbreak, while its rise time is not significantly different from the baseline scenario without any robotic implementation (Figure 2, red curve; T_10_ = 12.0 ± 0.7 days, p_val_ = 0.074, permutation test). Table 1 and Supplementary Table S3 contain more details on the temporal evolution of active infections for each robotic scenario.

**TABLE 1:** Measures of the temporal evolution of infection spreading for a baseline case of R_0_ = 4.4.

Scenario	R_0_ after intervention	Individuals infected	T10	I30
Reproductive number (R_0_ = 4.4)				
No robotic assistance	4.4 ± 0.3	71 ± 2.4	10.1 ± 0.7	32.5 ± 0.8
Assist rand 5 NUR	3.8 ± 0.3	63 ± 0.7	11.9 ± 0.8	26.8 ± 1.0
Assist top 5 NUR	2.7 ± 0.2	54 ± 2.9	17.1 ± 0.8	19.2 ± 1.0
Assist top 3 NUR-Rand 2 MD	3.2 ± 0.3	57 ± 3.0	14.2 ± 0.7	24.3 ± 0.9
Assist all NUR-PAT contacts	3.1 ± 0.2	47 ± 2.5	12.0 ± 0.7	23.8 ± 0.7

The measures consist of the effective R_0_ for each robotic implementation scenario, the total number of individuals infected by the end of the outbreak, the number of days until the 10th infection occurs (T_10_), and the number of active infections on the 30th day of the outbreak (I_30_).

### The Impact of the Basic Reproductive Number R_0_

**FIGURE 3:**
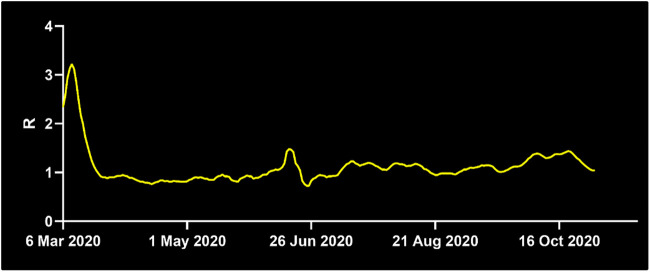
Development of the basic reproductive number R_0_ in Germany. While R_0_ was at 1.3 on April 7th, 2020, it decreased below the critical value of R_0_ = 1 shortly after April 7th until June 21st, where it surged to 2.03. For the majority of time thereafter, R_0_ stayed above 1, at the time of writing it is 1.22. Source: Reprinted with permission from Robert-Koch-Institute.

Another important factor for infectious spread prediction is the basic reproductive number R_0_. During the first wave of Covid-19, R_0_ ranged between 2 and 6 considering all countries (Sanche et al., 2020), values of 3.2–3.4 were reported for China (Alimohamadi et al., 2020), Austria, Switzerland (Karnakov et al., 2020), Italy, Korea (Zhuang et al., 2020) and Germany (Dehning et al., 2020). Measures including the use of personal protective equipment (PPE), social distancing and frequent sanitization have reduced the basic reproductive to values as low as 0.6 (Fisman et al., 2020). After investigating the role of robotic integration for pathogen spread, we analyzed how different reproductive numbers R_0_ affect these measures. To this end, we simulated the virus spread in the above-mentioned scenarios for different R_0_ values. R_0_ values around 3.15 were reported in a meta-analysis from China (He et al., 2020). R_0_ values around 1.1–1.2 are estimated after the first wave of Covid-19 infections (Karnakov et al., 2020) (Figure 3). Basic reproductive numbers between 1.2 and 0.6 have also been used to model the effect of using personal protective equipment, such as masks, depending on the compliance and viral reduction rate provided. Therefore, in the model, we have focused on fitting the basic reproductive numbers of 3.4, 1.2, and 0.6, respectively, and studied the impact of robotic implementation under each of these epidemiological conditions. For R_0_ = 3.4, the targeted scenarios of replacing high-risk nurses or the interactions between nurses and patients result in the largest non-spreading probability (see Table 2 and Supplementary Figure S1). These two scenarios also lead to the smallest number of infected individuals by the end of the outbreak. The impact of robots on R_0_ = 3.4 is thus qualitatively similar to that obtained when R_0_ = 4.4. On the other hand, the benefit of robotic integration is limited when R_0_ = 0.6 since the baseline case (i.e. no robotic intervention) already results in 92% of non-spreading trials. However, we note that in some robotic scenarios the pathogen propagation can be stopped in 100% of the trials. It is also important to note that when R_0_ < 1, each infected individual infects less than 1 other individual on average and therefore, pathogen spread is relatively well controlled. Interestingly, basic reproductive numbers near the critical value of 1, such as R_0_ = 1.2, provide the largest gain for robotic scenarios, even more than for larger R_0_ that we have considered (i.e., 3.4 and 4.4). The probability not to spread the virus increased from 67% at baseline to 96% with targeted robotic replacement. Overall, these results indicate that the qualitative effects of robotic scenarios are robust across a range of basic reproductive numbers with significant benefit occurring at regimes with a relatively large value of R_0_, and specially with R_0_ slightly above its critical value of 1.

**TABLE 2:** Results for the probability of pathogen spread across the network for different reproductive numbers.

Scenario	Reproductive number R_0_			
	4.4	3.4	1.2	0.6
Ratio of non-spreading trials				
No robotic assistance	0.1	0.22	0.67	0.92
Assist rand 5 NUR	0.16	0.2	0.83	1.0
Assist top 5 NUR	0.22	0.28	0.96	1.0
Assist top 3 NUR-Rand 2 MD	0.21	0.24	0.96	0.99
Assist all NUR-PAT contacts	0.23	0.27	0.75	0.96

## Discussion

Our model investigates whether robotic assistance to a moderate number of HCW influences the pathogen spread rate in a geriatric ward. We find that targeted robotic replacement of nurses with the largest number of personal close contacts results in the largest effect to control infectious spread. This scenario not only decreases the probability of viral spread but also slows down its outbreak. The most optimal R_0_ to integrate robots into clinical use is when the reproductive number is slightly higher than the critical value of R_0_ = 1, while significant effects are still observed for relatively high values (at least up to 4.4).

### Limitations and Shortcomings of the Model

The present model is limited in several aspects. First, it only considers pathogen spread due to close personal contact. However, growing evidence suggests that aerosol and fomite transmission are additional routes of infection of Covid-19 (Lu et al., 2020; Shen et al., 2020). Detailed models simulating the physics of aerosol and fomite transmission have been applied to hospital infrastructures during outbreaks of other diseases such as influenza (Kraay et al., 2018; Wong et al., 2010), and could be integrated to refine our basic model on the impact of robotic scenarios on pathogen spread. Robots such as for example those used as companion robots can be in frequent contact with several patients and thus also be a source of pathogen contagion *via* fomite transmission. Maintenance and cleaning of robots, including disinfection robots, are other potential sources of transmission for healthcare and maintenance personnel. These effects can also be modeled for an accurate balance of the effect of robotic integration in the spread of infectious diseases with surface contact as a major *via* of transmission. Second, our spreading model runs on real proximity contact data obtained from a geriatric unit during a non-pandemic state. While we considered a range of values of the reproductive number that occurred at different stages of the Covid-19 pandemics, it will be necessary to also test the model with contact data obtained at such stages. Third, detailed proximity contact data of HCW and patients are not publicly available for larger healthcare infrastructures such as entire hospitals. How the predictions of the effects of robotic assistance scale to larger nursing homes and geriatric units will require further data. Fourth, modeling a specific robotic system integration within the real network of proximity contacts would require a refined annotation of the activity conducted during each contact to determine which interactions can be replaced by specific types of robot and tasks. Our model abstracted from the specific robotic system including the network of specific personnel and patients, and assumed that robotic integration operated at the level of an effective number of node replacement or removal of interactions.

Overall, we believe that the present model is a step in modeling the potential impact of robotic assistance in pathogen spread, and that new data will make possible models tailored to specific situations and robotic systems.

### Yield, Timing and Cost of Robotic Integration Into Clinical Use

Robots have been utilized to assist humans in a variety of hazardous tasks, including visual aid (Xing et al., 2017) for firefighters (Tuffield and Elias, 2003), in nuclear environments and in mountain rescue (Sugiyama et al., 2013). The use of robots for infectious diseases has come into the spotlight with the Covid-19 pandemic. Acquisition of new robotic technology considers two major factors for hospital administrations: Yield and cost. Our study suggests that robots can significantly reduce infectious spread to protect healthcare workers and patients. Our data points out that the determination of HCW with a high number of contacts and targeted replacement of these HCW yields the most effective reduction in pathogen spread. While robots have been used during Covid-19 to employ an entire unit of a hospital (https://hbr.org/2020/04/how-hospitals-are-using-ai-to-battle-covid-19. https://www.medicaldevice-network.com/features/coronavirus-robotics/), our results demonstrate that integration of a smaller number of robots focused on high-risk HCW can significantly reduce cost and effectively decrease spreading probability. The basic reproductive number R_0_ also plays an important role in the efficiency of robotic implementation. During times, when R_0_ is below the critical number of 1, application of robots does not have a significant value, whereas with R_0_ values just above one or higher, robots can reduce infectious spread effectively. Thus, monitoring of R_0_ (Figure 3) and selection of appropriate time windows is a critical factor. Another important consideration is the use of personal protective equipment (PPE). PPE shortage has been a key concern during the Covid-19 pandemic, creating competition between governments and prioritizing certain countries over others. The integration of robots reduces the need for PPE since less HCW work within the unit. This can alleviate pressure during PPE shortage. In addition, robots can serve at maximum capacity to meet the increased need during extraordinary times.

## Methods

### Simulation of Infectious Spread

**FIGURE 4:**
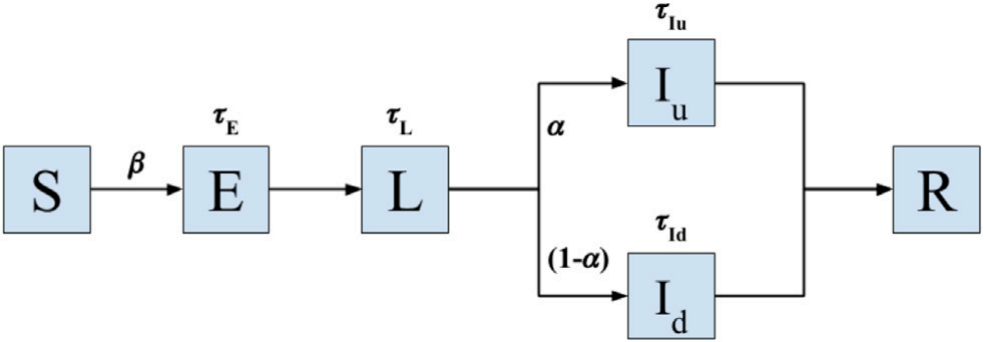
Model of Covid-19 transmission. Transition model used to simulate the spread of infectious disease with epidemiological characteristics of Covid-19 on a real sequence of contact data in a geriatric unit. Upon close contact with a latent (L) or infected case (I_u_ and I_d_ stand for infected asymptomatic and diseased states, respectively), an individual in susceptible state (S) will transition to the state of exposed (E) with probability β, which controls the transmissibility of the disease. The individuals in the E state remain in such a state for a time interval τE before entering into the latent state (L). After a duration τL an individual in the latent state will transition with probability α to an infected undetected (or asymptomatic) state (I_u_), and with probability 1-α to an infected diseased (I_d_) state. Each infected individual remains in its infected state for latencies τI_u_ and τI_d_, respectively, before being effectively removed from the spreading population after becoming removed or recovered (R). The model operates until all individuals belong to either the S or R states, the moment in which the local outbreak ends. All latencies are probabilistically sampled from Gamma distributions fitted for the dynamics of Covid-19 (Lauer et al., 2020).

For pathogen spread, we consider a model with six states according to the disease status of the individual: Susceptible (S), exposed (E), latent (L), infected undetected (I_u_), infected diseased (I_d_), and recovered (R) (Ivorra et al., 2020). See Figure 4 for the graphical depiction of the model and transitions between states. The model is a recent variation of the well-established Susceptible-Exposed-Infected-Recovered (SEIR) model, adapted to the epidemiological characteristics of Covid-19, including the latencies (Eguíluz et al., 2020; Flaxman et al., 2020; Bi et al., 2020; Lauer et al., 2020) and the reported large fraction of asymptomatic cases occurring in this disease (Ivorra et al., 2020). All individuals start with a susceptible status. Upon close contact with an infected or latent case, an individual in susceptible state (S) will transition to the state of exposed (E) with probability β, which controls the transmissibility of the disease. An individual will remain in the E state for a time interval of duration τE before entering into the latent state (L). After a duration τL an individual in the latent state will transition with probability α to an infected undetected (or asymptomatic) state (I_u_), and with probability 1-α to an infected diseased (I_d_) state. Each infected individual remains in its infected state for latencies τI_u_ and τI_d_, respectively, before being effectively removed from the spreading population after becoming removed or recovered (R).

Under different scenarios, we quantify the infectious spread occurring from a single individual. The model is based on a real sequence of close proximity contacts obtained from a geriatric unit as described in the section *Proximity contact dataset*.

The proximity contact network is critical to simulate the model in a real setting and avoid the assumption of random or homogeneous mixing of individuals in a population that is characteristic of models used at larger scales. As the propagation of Covid-19 takes longer than 4 days (duration of the recordings of the real sequence of close contacts), we repeat the interaction sequence in a loop when running the infectious spread model.

For each scenario simulated, we randomly select one nurse to become infected at day zero and run the spread model until all individuals eventually belong to the S or R state. That is, the spread and simulation stop when only susceptible and recovered individuals remain. We repeat each simulation 100 times to account for the variability of the spread dynamics (measured by the standard error of the mean; s.e.m.) and collect averaged statistics. The average computer running time for the 100 trials was 8 h per scenario on Google Collab (on machines that use single core hyper-threaded Xeon processors running at 2.3 GHz). The simulations are implemented in python as interactive python notebooks and can be found at the repository: https://github.com/Mo-youssef/Robotic-Impact-Simulation.git.

### Incubation Period and Latencies

The incubation period, which includes the E and L compartments, is estimated at around 6 days (Bi et al., 2020) Specifically, the incubation period considers an average latency of 2 days in exposed (E compartment) and 4 days in the latent state (L compartment), in which the infected person is undetected but still contagious. All latencies are randomly sampled from Gamma distributions (Flaxman et al., 2020). The Gamma distributions have a shape parameter of 3, and we fit their scale parameter to adjust the mean of the distribution to the reported values. All default model parameters are described in Supplementary Table S4.

### Proximity Contact Dataset

Infectious spread is modelled based on real data of personal contacts among patients, as well as between patients and HCW in a geriatric unit (Vanhems et al., 2013). The dataset was obtained using unobstructive wearable badges embedded with small active radiofrequency devices that could exchange ultra-low-power radio packets when facing another tag within a distance of 1.5 m. The dataset includes proximity contact data for 29 patients (PAT) and 46 HCW. Out of these 46 HCWs, there are 27 nurses (NUR), eleven doctors (MD) and eight administrators (ADM). A contact was recorded when two individuals faced each other for more than 20 s. The contacts were recorded during 4 days and four nights. There was a total of 14,037 contacts in this period, including contacts extending 20 s, for a total of 10,808 min of contact.

### Scenarios

We study the effect of robotic assistance by simulating four scenarios and comparing them to the case without robotics intervention.

In total, the five scenarios are:

1. No robotic assistance.
2. Assistance to five random nurses.
3. Assistance to Top 5 high-risk nurses.
4. Assistance to Top 3 high-risk nurses and 2 random medical doctors.
5. Assistance in all interactions between nurses and patients.

In all cases, it is assumed that the robotics assistance allows interactions of the specific HCW being assisted to not be of a close-contact type and hence, to not lead to contagion by personal proximity with an infected individual.

### Measures Used to Characterize the Infectious Spread

**R**_**0**_ **.** R_0_ is the basic reproduction number and is calculated as the average number of people who are directly infected by one person with the disease.

**Number of susceptible cases.** It refers to the number of subjects remaining in the susceptible state, i.e. who have not been infected by the end of the outbreak episode.

**Propagating trials.** We define the infection dynamics as propagating when the spread is such that the fraction of susceptible individuals by the end of the episode is less than 90% of the total population. For the scenarios described above, we report the fraction of trials in which the spread dynamics are propagating and the average percentage of infected individuals.

**I**_**30**_ **.** I30 is calculated as the number of active infection cases (individuals in states E, L, I_u_ or I_d_) on day 30 after the first infection among the population.

**T**_**10**_ **.** T_10_ is the number of days elapsed from the first to the 10th infection in the population.

In all cases, we always report the values for these metrics as their mean value over 100 trials together with their respective standard error of the mean.

### Calibrating the Probability of Disease Transmission Upon Proximity Contact

The probability of disease transmission of disease upon close contact (*β*) is the main parameter controlling the spread of infection in the model. To determine a reasonable estimate for this parameter, we scanned a range of values of *β* from 0.0001 to 0.0095 at steps of 0.001. For each value of *β*, we repeat 10 simulations of the model for 50 days to determine the basic reproductive number. We determine that the range of *β* between 0.0001 and 0.0025 results in spreading simulations displaying a basic reproductive number R_0_ in the most frequent ranges reported along the pandemic for Covid-19 (Flaxman et al., 2020).

## Data Availability

The original contributions presented in the study are included in the article/Supplementary Material, further inquiries can be directed to the corresponding authors.
