# Supplementary material for: Modelling the Impact of Robotics on Infectious Spread Among Healthcare Workers
Source: Front Robot AI. 2021 May 25;8:652685. doi: 10.3389/frobt.2021.652685 (PMC8185357; doi:10.3389/frobt.2021.652685)
Supplement: Supplementary file 1 [file DataSheet1.pdf]

frontiers

Supplementary Material

1. Supplementary Figures and Tables

Roboter Name	Function	Company	Literature	Source
	Introducing needles and catheters into deformable tissues such as blood vessels to draw blood or deliver fluids by using ultrasound	Department of Biomedical Engineering, Rutgers University of New Jersey	Chen et al. (*1*)	
**SleepSmart**	Multi-sensor mattress pad to diagnose chronic sleep disorders by detecting data such as heart rate, breathing rate, body orientation and index of restlessness		Van der Loos et al. (*2*)	
**Morpheus**	Mattress actuation system to encourage a person to roll over in bed to alleviate snoring based on acoustic sensor data analysis			
	Measuring vital signs	University of Auckland, New Zealand	Broadbent et al. (*3*)	
**ElliQ**	Social tasks: virtual communication, proactive suggestions, entertainment and health activities	Intuition Robotics		
	Organizational tasks: medication reminders, apointment scheduling, cab calls			
**ROBEAR**	Lifting and carrying patients (nursing robot): elderly and handicapped	RIKEN and Sumitomo Riko Limited		
**RIBA / RIBA-II**	Lifting and carrying patients (nursing robot)	RIKEN		https://

**SAM**	remote monitoring, physical safety (preventing falls), and virtual communication	Luvozo		roboticsbiz.com/top-seven-companion-and-social-robots-for-elderly-people/
**Zora**	Interactive caregiver: leading physical therapy classes, activities and exercises, entertaining patients (reading, TV etc.), educational tasks	Zora Bots / Oz Robotics		
**iPal**	Caregiver: social companion, educator, safety monitor, health and emergency service and virtual communication	Avatar Mind		
**Care-O-Bot**	Household tasks: delievering food, drinks and medications to patient, assisting in cooking and cleaning	Fraunhofer Institute for Manufacturing Engineering and Automation		
	Health care: emergency calls, virtual communication			
**BUDDY**	Detection of falls or unusual events, alert emergency service, home protection, tracking capabilities	Blue Frog Robotics		
**Xenex Germ-Zapping Robot**	Disinfection of entire hospital rooms within minutes using pulsed, full-spectrum UV rays that kill a range of infectious bacteria and viruses	XENEX Disinfection Systems		https://online-engineering.case.edu/blog/medical-robots-making-a-difference
**PARO Therapeutic Robot**	An animal-like robot that provides the benefits of animal therapy as a treatment for depression or other mental illness or as a method to recover better from surgery	National Institute of Advanced Industrial Science and Technology (AIST)		
**TUG**	Ferrying supplies such as meals, linens, lab samples, waste and other items/materials	Aethon		

**GermFalcon**	Cleaning robot using UVC light to kill viruses and bacteria	Dimer UVC Innovations		https://www.medicaldevice-network.com/features/coronavirus-robotics/

Table S1. **Examples of assistive medical robots used in various fields of healthcare.**

**Reproductive number ($R_0 = 4.4$)**			
	**Non-spreading**	**Infected individuals**	
**Scenario**	**Ratio of non-spreading trials**	**Mean % of infected individuals**	**Median % of infected individuals**
No robotic assistance	0.10	71	78
Assist Rand 5 NUR	0.16	66	76
Assist Top 5 NUR	0.22	57	71
Assist Top 3 NUR-Rand 2 MD	0.21	60	71
Assist all NUR-PAT contacts	0.23	47	57

Table S2. Effect of robotic interventions in different scenarios. Percentage of non-spreading trials, mean and median percentage of infected individuals in the geriatric. Results for $R_0 = 4.4$ over 100 trials.

**Reproductive number ($R_0 = 4.4$)**				
	**I_{30} (number of active infections at day 30)**			
**Scenario**	**NUR**	**MD**	**PAT**	**ADM**
No robotic assistance	11.8 ± 0.4	6.8 ± 0.2	11.3 ± 0.3	2.7 ± 0.1
Assist Rand 5 NUR	9.0 ± 0.4	6.2 ± 0.3	9.2 ± 0.4	2.6 ± 0.2
Assist Top 5 NUR	6.7 ± 0.3	5.2 ± 0.4	5.5 ± 0.4	2.0 ± 0.2
Assist Top 3 NUR-Rand 2 MD	9.4 ± 0.4	4.4 ± 0.3	8.1 ± 0.4	2.5 ± 0.1
Assist all NUR-PAT contacts	10.4 ± 0.4	6.6 ± 0.3	4.3 ± 0.3	2.7 ± 0.1

Table S3. Splitting of infectious cases on day 30 (I_{30}) by category. Results for $R_0 = 4.4$ over 100 trials.

**Parameter**	**Description**	**Value**
β	Probability of disease transmission upon contact with Latent or Infected individual	0.0015
α	Proportion of asymptomatic infections	0.8
τ_E	Average latency from Exposed to Latent	2 days
τ_L	Average latency from Latent to Infected	5 days
τ_{Iu}	Average latency from Asymptomatic Infected to Recovered	12 days
τ_{Id}	Average latency from Symptomatic Infected to Recovered	7 days

Table S4. Default model parameters.

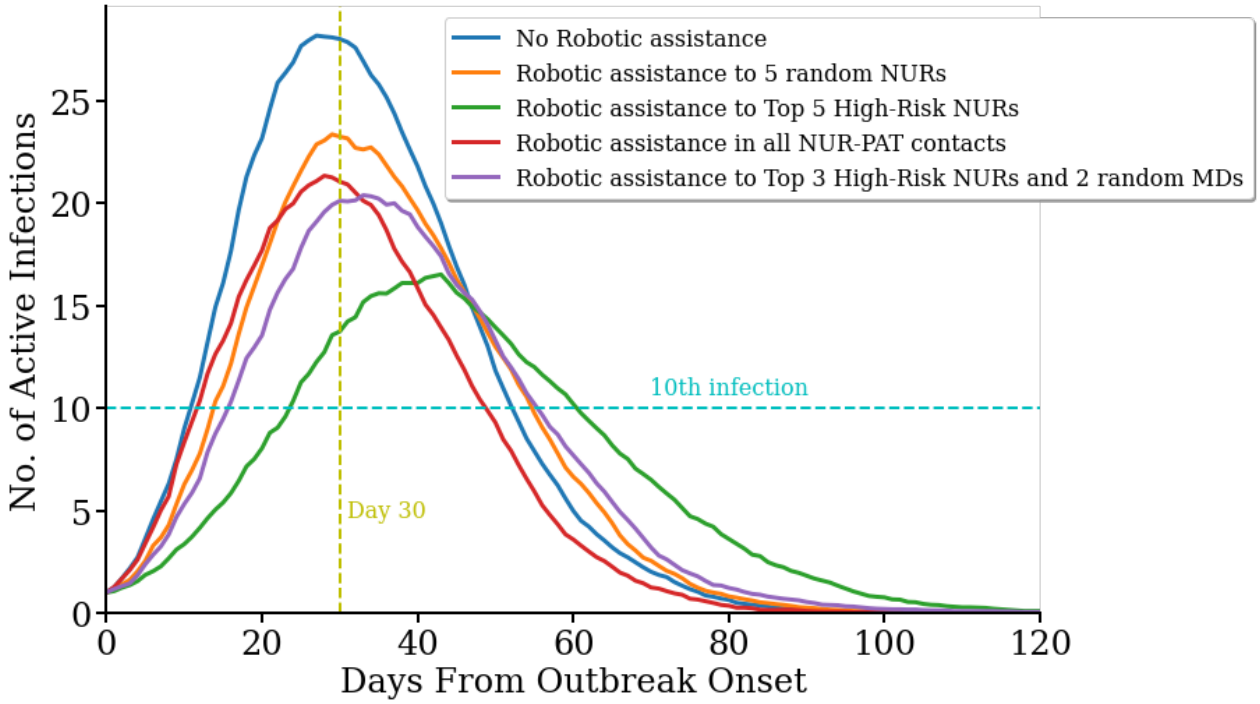

Figure S1. Temporal evolution of infection spread across the network for different scenarios. R_0 = 3.4. Number of active infected cases in 5 different scenarios as a function of the days passed since the first infection in the population at day 0. Average over 100 trials.

References for Supplementary Information:

1. A. I. Chen, M. L. Balter, T. J. Maguire, M. L. Yarmush, Deep learning robotic guidance for autonomous vascular access. *Nat. Mach. Intell.* (2020), doi:10.1038/s42256-020-0148-7.
2. H. F. Machiel Van der Loos, N. Ullrich, H. Kobayashi, Development of sensate and robotic bed technologies for vital signs monitoring and sleep quality improvement. *Auton. Robots* (2003), doi:10.1023/A:1024444917917.
3. E. Broadbent, J. R. Orejana, H. S. Ahn, J. Xie, P. Rouse, B. A. Macdonald, in *Proceedings - IEEE International Workshop on Robot and Human Interactive Communication* (2015).
